# Neural alterations of emotion processing in atypical trajectories of psychotic-like experiences

**DOI:** 10.1038/s41537-022-00250-y

**Published:** 2022-04-21

**Authors:** Roxane Assaf, Julien Ouellet, Josiane Bourque, Emmanuel Stip, Marco Leyton, Patricia Conrod, Stéphane Potvin

**Affiliations:** 1 Centre de Recherche de l’Institut Universitaire en Santé Mentale De Montréal, Montreal, QC Canada; 2 Department of Psychiatry and Addiction, Faculty of Medicine, University of Montreal, Montreal, QC Canada; 3 Centre de Recherche du Centre Hospitalier Universitaire Sainte-Justine, Montreal, QC Canada; 4 Department of Psychiatry, Perelman Faculty of Medicine, University of Pennsylvania, Philadelphia, PA USA; 5 Department of Psychiatry, Faculty of Medicine, McGill University, Montreal, QC Canada

**Keywords:** Prefrontal cortex, Psychosis

## Abstract

The aim of this study was to investigate the neural bases of facial emotion processing before the onset of clinical psychotic symptoms in youth belonging to well-defined developmental trajectories of psychotic-like experiences (PLEs). A unique sample of 86 youths was recruited from a population-based sample of over 3800 adolescents who had been followed from 13 to 17 years of age. Three groups were identified based on validated developmental trajectories: a control trajectory with low and decreasing PLEs, and two atypical trajectories with moderate to elevated baseline PLEs that subsequently decreased or increased. All had functional magnetic resonance imaging data collected during a facial emotion processing task. Functional activation and connectivity data were analyzed for different contrasts. The increasing PLE trajectory displayed more positive psychotic symptoms while the decreasing trajectory exhibited more negative symptoms relative to the control group. During face processing, both atypical trajectories displayed decreased activations of the right inferior frontal gyrus (IFG), while the increasing trajectory displayed a negative signal in the precentral gyrus. The increasing PLE trajectory also displayed impaired connectivity between the amygdala, ventromedial prefrontal cortex, and cerebellum, and between the IFG, precuneus, and temporal regions, while the decreasing trajectory exhibited reduced connectivity between the amygdala and visual regions during emotion processing. Both atypical PLE trajectories displayed alterations in brain regions involved in attention salience. While the increasing trajectory with more positive symptoms exhibited dysconnectivity in areas that influence emotion salience and face perception, the decreasing trajectory with more negative symptoms had impairments in visual information integration areas. These group-specific features might account for the differential symptom expression.

## Introduction

Psychotic disorders affect 0.31% of the US population^1^ and are associated with an average of 14.5 years of potential life lost^2^. The first clinical signs are commonly observed during late adolescence, a critical developmental period characterized by cognitive and emotional maturation, and marked neuroanatomical and neurofunctional reorganization^3^. While diverse neurodevelopmental abnormalities (e.g., birth complications, soft neurological signs, neurocognitive deficits, and neurodevelopmental disorders) have been associated with risk for psychotic disorders, few have been directly and specifically linked to psychosis. The study of Psychotic-Like Experiences (PLEs) offers a unique opportunity to investigate more specific processes.

Psychosis is thought to exist on a continuum^4^ with, at one end, schizophrenia, a mental disorder characterized by delusions, hallucinations, disorganized speech, and behavior; an intermediate treatment-seeking Clinical High Risk syndrome characterized by “attenuated” or “brief” symptoms that do not necessarily meet full diagnosis criteria; and, at the other end, PLEs. PLEs are defined as subclinical psychotic symptoms that can be observed in the general population, including 17% of children aged 9–12 and over 7% of adolescents^5^.

While most PLEs are transient in nature and do not extend into adulthood, persistent PLEs confer a four- to ten-fold increased risk for psychosis^6–8^. Recent cohort studies provide evidence of heterogeneous PLE developmental trajectories during adolescence. For example, in 2566 youths from the general population and followed between the ages of 13–16, three trajectories were identified: 83.9% of adolescents exhibited a typical trajectory of low PLE levels that decreased further over time, 7.9% exhibited high PLE levels that subsequently decreased, and 8.3% reported moderate PLE levels that increased^9^. These trajectories have been consistently replicated in separate samples^10–12^, which supports their relevance in adolescent development. While the increasing trajectory is thought to be associated with increased risk of psychosis^13^, outcomes in the decreasing trajectory have not been investigated. Furthermore, the neural correlates of these well-documented trajectories are unknown. Studying PLEs and their developmental trajectories provides a unique opportunity to identify clinical and neural correlates of psychosis vulnerability without the confounding effects of antipsychotic medication and comorbidities.

Socio-emotional dysfunction is considered a hallmark of psychotic disorders. It is manifested by deficits in emotion perception, responsivity, and expression, and is strongly associated with impaired social functioning in schizophrenia patients^14^. The neural correlates underlying these deficits have been well-documented^15^ and include impaired limbic activations^15,16^ and decreased amygdala–prefrontal connectivity^17^ during facial emotion processing.

Some studies in youth reporting PLEs have found increased limbic responses to ambivalent faces^18^ or to threatening faces^19^, while others have observed decreased responses to emotional faces in the prefrontal, parahippocampal, and striatal regions^20^. In addition to their association with local activity changes, PLEs were associated with decreased amygdala–prefrontal connectivity during negative emotion processing^21^. These findings suggest that functional alteration of frontolimbic regions during the processing of emotional content may constitute a marker of a broad vulnerability to psychosis. However, no study has integrated knowledge on the heterogeneity of PLE trajectories to examine potential neural differences in emotion processing.

The current study aimed to investigate whether youth belonging to distinct developmental PLE trajectories can be differentiated on clinical and socio-emotional information processing indices. To our knowledge, this is the first study to investigate the neural correlates of emotion processing in youth belonging to well-defined PLE developmental trajectories. Participants were a unique general population sample selected from over 3900 adolescents characterized on population-based data-driven latent developmental trajectories and being followed for 10 years. We hypothesized that those following an increasing trajectory of PLEs would show altered activity and connectivity patterns in limbic and prefrontal regions involved in emotion processing and salience, respectively. We also hypothesized that the decreasing trajectory would present distinct clinical and neural correlates compared to the control and increasing trajectory.

## Results

### Demographic and clinical variables

Demographic and clinical variables for each of the three trajectories are reported in Table 1. No significant group differences were detected for age, gender, or handedness. While past year cannabis and tobacco use were not different between groups, alcohol use was significantly higher in PLE-1 (decreasing) than PLE-2 (increasing) (Table 1). Therefore, all following analyses controlled for past year alcohol use. Past month substance use, assessed by timeline-followback interview, showed no group differences (data not shown).

**Table 1:** Demographic and clinical characteristics.

	PLE-0 (*N* = 41)	PLE-1 (*N* = 19)	PLE-2 (*N* = 26)	Statistics
Demographic				
Age (SE)	17.22 (0.11)	17.37 (0.19)	17.31 (0.17)	*F* = 0.25; *p* = 0.78
Gender, % male	46.3	42.11	53.84	*χ*^2^ = 0.66; *p* = 0.72
Handedness, % right-handed	97.56	94.74	96.15	*χ*^2^ = 0.851; *p* = 0.85
Substance use (12 m)				
Cannabis use level (SE)	0.87 (0.21)	1.5 (0.41)	1.3 (0.18)	*F* = 1.18; *p* = 0.313
Alcohol use level (SE)	2 (0.16)	**2.61**^**c**^ (0.20)	**1.56**^**c**^ (0.23)	***F*** = **5.68;** ***p*** = **0.005**
Tobacco use level (SE)	0.76 (0.25)	1.39 (0.40)	0.74 (0.36)	*F* = 1.05; *p* = 0.356
PLE				
Total score (SE)	**0.46**^b^ (0.14)	**0.74**^**c**^ (0.24)	**2.17**^**b,c**^ (0.50)	***F*** = **9.53;** ***p*** < **0.001**
CAPE-42				
Depression scale (SE)	5.29 (0.67)	6.79 (0.83)	6.83 (0.73)	*F* = 1.58; *p* = 0.214
Positive scale (SE)	**4.49**^**b**^ (0.69)	5.53 (0.87)	**8**^**b**^ (1.12)	***F*** = **4.37;** ***p*** = **0.016**
Bizarre thoughts (SE)	**1.57**^**b**^ (0.33)	1.84 (0.47)	**3.56**^**b**^ (0.66)	***F*** = **5.14;** ***p*** = **0.008**
Delusional thoughts (SE)	2.91 (0.45)	3.68 (0.51)	4.43 (0.57)	*F* = 2.44; *p* = 0.094
Negative scale (SE)	**7.6**^**a**^ (1.02)	**12.05**^**a**^ (1.79)	9.87 (1.24)	*F* = 2.30; *p* = 0.056
Total score (SE)	17.37 (2.01)	24.37 (3.00)	24.7 (2.78)	*F* = 3.09; *p* = 0.051
DAWBA diagnosis				
Generalized anxiety (SE)	1.25 (0.17)	1.78 (0.22)	1.83 (0.23)	*F* = 2.80; *p* = 0.067
Depression (SE)	1.12 (0.24)	0.72 (0.18)	1.54 (0.35)	*F* = 1.78; *p* = 0.175
Emotional disorder (SE)	0.29 (0.11)	0.28 (0.13)	0.42 (0.15)	*F* = 0.33; *p* = 0.722
Behavioral disorder (SE)	0.06 (0.04)	0.17 (0.12)	0.12 (0.09)	*F* = 0.50; *p* = 0.609
Psychotic experiences total score (SE)	0.25 (0.13)	0.22 (0.13)	1.00 (0.34)	***F*** = **3.91;** ***p*** = **0.024**
ER-40 performance				
Average accuracy, % (SE)	85.38 (0.92)	84.86 (1.45)	84.5 (1.12)	*F* = 0.18; *p* = 0.832
Average RT, ms (SE)	2094.88 (52.19)	1875.69 (70.05)	2148.34 (80.43)	*F* = 3.58; *p* = 0.033

Bold values indicate results that are statistically significant between groups.

*SE* standard error; substance use rated on a Likert scale; *CAPE* Community Assessment of Psychotic Experiences, rated on a Likert scale, *PLE* psychotic-like experience score as determined by a 9-item questionnaire, *DAWBA* The Development and Well-Being Assessment, *ER-40* Emotion Recognition task (40 items).

^**a**^Significant difference between PLE-0 and PLE-1.

^**b**^Significant difference between PLE-0 and PLE-2.

^**c**^Significant difference between PLE-1 and PLE-2.

PLE scores at age 17 were significantly higher in the PLE-2 group compared to PLE-0 and PLE-1 (Table 1). Similarly, psychotic experiences, as measured with the DAWBA total scores, were significantly higher in PLE-2 compared to PLE-0 and PLE-1. The Community Assessment of Psychic Experiences (CAPE) positive symptom scale and bizarre thoughts subscale were significantly higher in PLE-2 compared to the PLE-0. In addition, a significantly higher score on the CAPE negative symptoms scale was observed in PLE-1 compared to PLE-0. No group differences were found for generalized anxiety, depression, emotional, and behavioral disorders. At this first timepoint, no participant had transitioned to a clinical diagnosis of a psychotic disorder. Finally, no significant group differences were found in ER-40 response accuracy and reaction time at the level of each emotion type nor at the level of average scores (Table 1).

### Brain activation results

**Fig. 1:**
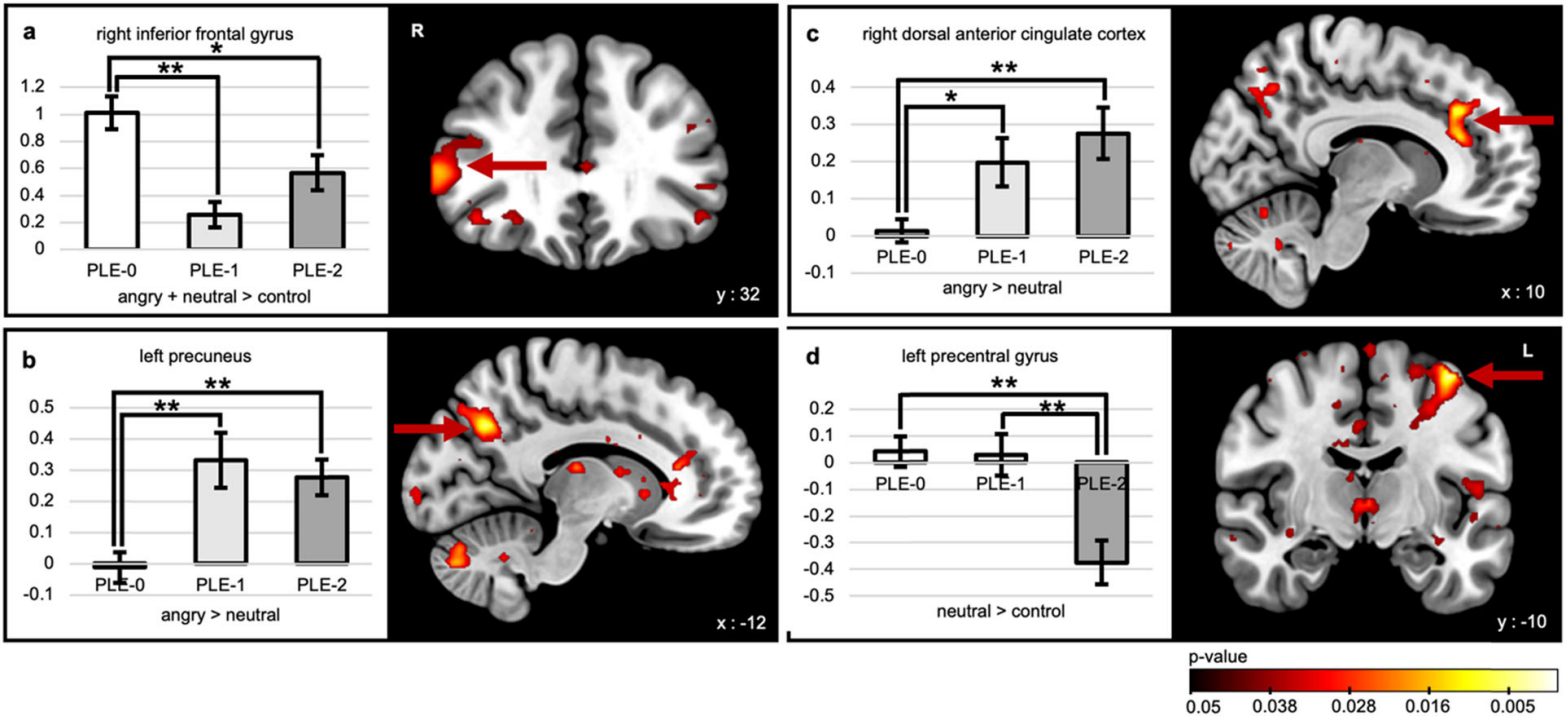
Differences in brain activation during emotion processing in the PLE trajectories compared to typically developing adolescents. Between-group brain activation results for the different contrasts and bar graphs of the corresponding beta values for the three trajectories (PLE-0 = control trajectory; PLE-1 = decreasing trajectory; PLE-2 = increasing trajectory): **a** the right inferior frontal gyrus in the [(Angry + Neutral) – Control] facial cue processing contrast, **b** the left precuneus, and **c** the right dorsal anterior cingulate cortex in the (Angry − Neutral) negative emotion processing contrast, **d** the left precentral gyrus in the (Neutral − Control) neutral facial processing contrast. *p < 0.05, **p < 0.005 (for display purposes, we used a peak threshold of *p* = 0.05).

For all contrasts, results are described in Table 2. During facial cue processing [(Angry+Neutral)–Control], the right inferior frontal gyrus (IFG) was hypoactivated in PLE-1 and PLE-2 (Fig. 1a) relative to the control group. During negative emotion processing (Angry−Neutral), the left precuneus and the right dorsal anterior cingulate cortex (dACC) displayed increased activation in PLE-1 and PLE-2 compared to the controls (Fig. 1b, c)—but this was statistically significant only when using a more liberal threshold. During neutral facial processing (Neutral−Control), the left precentral gyrus was found to be under-recruited in the PLE-2 group compared to PLE-0 and PLE-1 (Fig. 1d). Finally, for all contrasts, the ROI analyses revealed no between-group differences in amygdala activations.

**Table 2:** Between-group activation differences.

Contrast	Region	Peak coordinates			Peak *F* value	Cluster size	Peak threshold
		*X*	*Y*	*Z*			
(Angry + Neutral) − Control	Right inferior frontal gyrus	54	34	8	9.65	38	*p* < 0.001
Angry−Neutral	Right dorsal anterior cingulate cortex	12	32	32	10.42	89	*p* < 0.005*
	Left precuneus	−14	−64	34	9.84	59	*p* < 0.005*
Neutral−Control	Left precentral gyrus	−40	−10	62	14.97	42	*p* < 0.001

This table shows the results of the whole-brain activation analysis for the different contrasts, with the corresponding peak region names, their MNI coordinates, peak *F* value, cluster size, and the peak threshold used.

* With the more liberal peak threshold of 0.005, a cluster threshold of 31 voxels was used, as calculated with the Monte Carlo simulation.

### Connectivity analyses

Seed selection was based on the group differences brain activation results in each of the contrasts. Spheres were created around the peak coordinates of the right IFG, the left precentral gyrus, the left precuneus and the right dACC (radius of 6 mm for all regions, except for a radius of 10 mm for the dACC). The bilateral amygdala were also used as seed regions^22^.

**Fig. 2:**
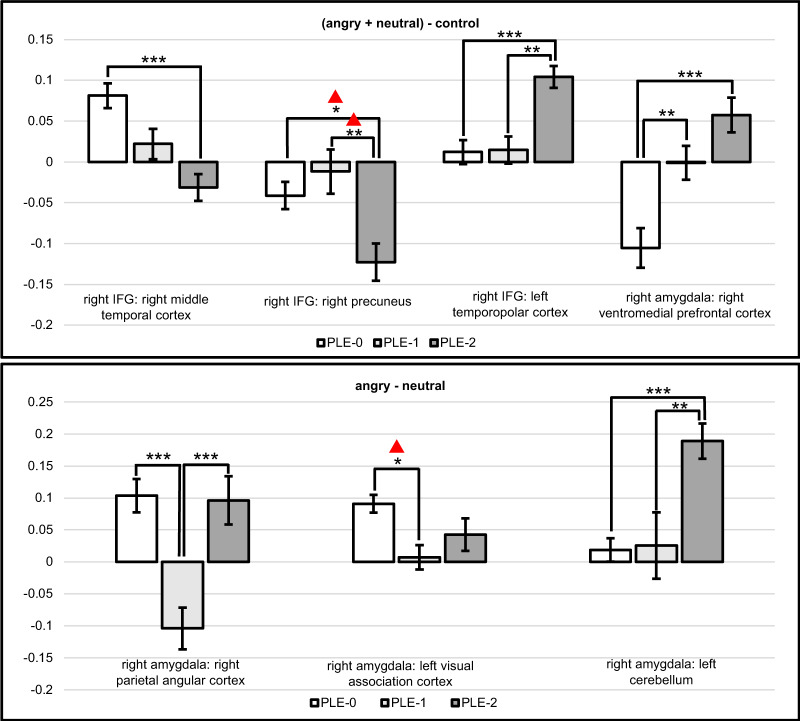
Connectivity analyses with the right IFG and right amygdala as seeds. Bar graphs showing the between-group functional connectivity results. The bar graphs show the beta connectivity scores for the [(Angry + Neutral) – Control] facial cue processing contrast and the (Angry − Neutral) negative emotion processing contrast for the three trajectories (PLE-0 = control trajectory; PLE-1 = decreasing trajectory; PLE-2 = increasing trajectory). *p < 0.05, **p < 0.01, ***p < 0.001. Not significant after applying a Bonferroni correction for the number of seeds (p < 0.002).

Functional connectivity differed between groups (Table 3 and Fig. 2). During facial cue processing [(Angry+Neutral)–Control], the PLE-2 group exhibited hypoconnectivity between right IFG and the right middle temporal gyrus, and between the right IFG and the right precuneus. However, this latter result did not remain significant after applying a Bonferroni correction for the number of seeds being tested. Conversely, PLE-2 exhibited hyperconnectivity between the right IFG and left temporopolar cortex relative to the other two groups. In both PLE-1 and PLE-2, compared to PLE-0, connectivity was increased between the right amygdala and medial prefrontal cortex (e.g., ventromedial prefrontal cortex, vmPFC).

**Table 3:** Between-group connectivity differences.

Contrast	Seed	Region	Region coordinates			Peak *F* value	Cluster size
			*x*	*y*	*z*		
(Angry + Neutral) − control	Right inferior frontal gyrus	Right middle temporal gyrus (BA 21)	48	−22	−10	12.64	80
		Right precuneus (BA 7)	6	−52	58	12.31	64
		Left temporopolar cortex (BA38)	−42	14	−38	14.16	50
	Right amygdala	Right ventromedial prefrontal cortex (BA 10)	8	58	−2	9.63	36
Angry−Neutral	Right amygdala	Right parietal angular cortex (BA 39)	32	−68	30	10.23	58
		Left visual association cortex (BA 18)	−32	−78	4	12.31	501
		Left cerebellum (CRUS-1 lobule)	−42	−76	−26	13.29	53

This table shows the results of the seed-to-voxel functional connectivity analysis for the different contrasts. The names of the selected seeds are presented with the corresponding voxels and their MNI coordinates, peak *F* value, and cluster size.

*BA* Brodmann area.

During negative emotion processing (Angry−Neutral), PLE-1 showed hypoconnectivity between the right amygdala and the left visual association cortex compared to PLE-0; however, this effect was not significant at the Bonferroni-corrected *p* value. The decreasing trajectory also displayed hypoconnectivity between the right amygdala and the right parietal angular cortex relative to the two other groups. In comparison, PLE-2 exhibited increased connectivity between the right amygdala and the left cerebellum Crus I lobule relative to the two other groups.

### Correlations with psychotic symptoms

Across groups, right amygdala to left cerebellum connectivity positively correlated with CAPE positive bizarre thinking score during negative emotion processing (Angry−Neutral) (*r* = 0.326; *p* = 0.004). This finding was near significance using a Bonferroni-corrected *p* value of 0.001. Other CAPE scales did not significantly correlate with brain activations.

## Discussion

The current study highlights the relevance of differentiating between developmental trajectories of PLEs when attempting to understand psychosis-proneness. At the clinical level, the PLE group with initially elevated but subsequently declining PLE scores exhibited more negative symptoms at 17 years of age, whereas those who reported progressively increasing PLEs reported more positive symptoms of psychosis and bizarre thoughts. This distinction is in line with evidence that increasing PLEs are associated with a higher risk of psychosis. It also adds to preliminary evidence that the decreasing trajectory is associated with a different symptom profile that includes fewer positive symptoms, but risk developing a psychiatric disorder nonetheless^23^. While we did not find group differences in clinical depression levels, the increased negative psychotic symptoms as measured by the CAPE-42 could reflect subclinical signs of depression. In fact, negative psychotic symptoms have been shown to be associated with depressive symptoms in youths^24^. At the neural level, we showed that atypical PLE trajectories are associated with abnormal activity in the precuneus, precentral gyrus, dACC, and IFG during processing of a socio-emotional task. The decreasing trajectory further presented with amygdala–visual cortex hypoconnectivity, while the increasing trajectory was associated with altered connectivity between the IFG and precuneus and temporal regions, as well as between the amygdala and the vmPFC and cerebellum.

These findings of general and trajectory-specific neural activation patterns during emotion processing might help to explain heterogeneity of findings in the literature. First, during facial cue processing, both atypical trajectories exhibited hypoactivation of the right IFG. This is in line with right IFG hypoactivation during facial emotion processing in schizophrenia^25^. The right IFG is thought to be involved in higher-order executive functions such as cognitive control and attention allocation (e.g., filtering out irrelevant information)^26^. In the specific context of emotion processing, the region plays a critical role in emotion regulation^27^. However, since IFG hypoactivation was observed during facial cue processing (but not during negative processing), this result is more likely indicative of abnormal filtering of facial/social information, rather than emotional valence. Second, during negative emotion processing, both atypical trajectories showed a hyperactivation of the right dACC and left precuneus—brain regions that are involved in salience attribution^28^ and self-attribution^29^, respectively. Therefore, their increased activation could reflect increased recruitment of attentional resources and self-attribution of negative emotional stimuli—potentially reflecting an over attribution of negative valence to the face cue.

Altered activations in right IFG, right dACC, and left precuneus were found in both decreasing and increasing trajectories, meaning that these alterations are not trajectory-specific. In comparison, during neutral facial processing, a negative signal in the left precentral gyrus was specifically observed in the increasing trajectory, whereby this region was hyperactivated during the opposite contrast (Control−Neutral). The precentral gyrus encompasses the primary motor and premotor cortex and is thought to contain “mirror neurons” that are activated when viewing and understanding actions performed by others^30^ and is important for the motor preparation processes during the response to another’s intention. The fact that we captured the response to *dynamic* emotional expressions (as opposed to static images) in the fMRI task strengthens this interpretation. Consistent with findings in schizophrenia^16^ this suggests that the neural mechanisms involved in neutral facial processing are impaired in those at a higher risk of psychosis in the increasing trajectory. Similarly, activation alterations during neutral facial processing at age 14 has predicted psychotic symptoms at 2-year follow-up^18^, indicating that the processing of neutral faces, which is often overlooked in neuroimaging studies, may differentiate youths at risk of psychosis.

Connectivity analyses highlighted significant differences between PLE trajectories. In the increasing trajectory, during facial cue processing, we noted amygdala-vmPFC hyperconnectivity. The vmPFC is involved in fear-associated learning; therefore, this result may indicate increased salience attribution or aversive conditioning to facial cues^31^ which has also been observed in clinically confirmed psychotic disorders^32^. Interestingly, altered amygdala–prefrontal connectivity has been reported in adolescents at Clinical High Risk^33^, and in youths on the psychosis spectrum^34^. The aberrant amygdala–prefrontal connectivity over time was shown to correlate with an increased severity of positive symptoms, suggesting that abnormal neurodevelopment of the amygdala could be involved in the development of psychotic symptoms^34^. While both decreasing and increasing trajectories displayed altered activity of right IFG and left precuneus, only the increasing trajectory exhibited a hypoconnectivity between the IFG and precuneus. Additionally, this trajectory displayed abnormal connectivity between the right IFG and the lateral temporal pole and the middle temporal gyrus, which are known to be involved in the processing of visual social cues^35^ and decoding facial features^36^, respectively. Therefore, the increasing trajectory appears to exhibit alterations in coupling between frontal and temporal regions playing a key role in face processing. Finally, the increasing trajectory also exhibited amygdala–cerebellum hyperconnectivity during negative emotion processing, specifically within the nonmotor Crus I cerebellar lobule, which correlated with levels of bizarre thinking. This result is consistent with evidence showing that schizophrenia is associated with functional and structural changes within the Crus I lobule^37^, and propositions that complex interactions between cognition and emotion are involved in delusion formation^38^. The decreasing trajectory was also shown to be uniquely associated with hypoconnectivity between the amygdala and regions of the extended visual system (e.g., parietal and occipital regions). This feature might reflect a lack of integration of basic visual information during the early processing of emotional stimuli, leading to the aberrant response to emotional stimuli via bottom-up mechanisms^39^ potentially explaining the trajectory’s susceptibility to negative symptoms in young adulthood.

Despite the novel and robust research design of the Pro-Venture cohort, this study is not without limitations. The sample sizes of the atypical PLE trajectories were limited due to the low prevalence of decreasing and increasing trajectories in the general population and the difficulty in recruiting these participants into a neuroimaging study during young adulthood. The modest sample size could also account for the absence of differences on behavioral measures of emotion recognition and limbic system activation, and why dACC and precuneus activation effects were only evident using a more liberal threshold. Additionally, effective connectivity was not explicitly measured in this study, and therefore the directionality of altered connections cannot be determined. Nevertheless, this study successfully recruited a unique sample of psychosis-prone youths from a large population sample that was followed over 5 years, and it highlighted neural alterations that are specific to different PLE trajectories.

The current study showed that youth in atypical PLE trajectories exhibit differential clinical outcomes and altered neural processing of emotion stimuli. While both atypical trajectories displayed impaired activations of brain regions involved in information filtering during face processing, the current results distinguished between increasing and decreasing PLE developmental trajectories. The increasing trajectory displayed alterations in frontolimbic regions involved in aversive conditioning, which is consistent with the aberrant emotion salience hypothesis of psychosis^40^, and frontotemporal regions involved in face perception. The decreasing trajectory exhibited amygdala–visual cortex dysconnectivity potentially involved in the early processing of visual emotional stimuli. Some of the key findings emerged from the facial cue processing contrast as opposed to the emotion processing contrast, highlighting the importance of investigating both types of deficits. These findings also suggest that the increasing and decreasing PLE trajectories should be considered when studying risk profiles for psychosis and when attempting to understand the neurodevelopmental markers of risk.

## Methods

### Study design and participants

The ongoing Pro-Venture study (Prodromal psychosis in the Co-Venture study) is a 5-year longitudinal neuroimaging study that follows adolescents who showed one of three different PLE trajectories over a previous 5-year period during early adolescence. It is a neuroimaging add-on to the larger Co-Venture study, a cluster-randomized controlled trial evaluating the effectiveness of personality-targeted interventions on substance use^41^. A total of 3966 Grade 7 students from 31 high schools in the greater Montreal area were part of the Co-Venture cohort as they completed annual clinical assessments during class time from 13 to 17 years of age. Using latent class modeling with the 4-year data from ages 13 to 16, as described in the Supplementary Materials, three developmental trajectories of PLEs were identified among the total sample: a low-decreasing trajectory (control group, PLE-0), a high-decreasing trajectory (decreasing group, PLE-1), and a moderate-increasing trajectory (increasing group, PLE-2)^42^.

The Pro-Venture subcohort consists of 86 adolescents aged 16–20 at entry (mean age 17.22, SD = 0.72; 52.3% girls). Participants from the three PLE trajectories who had consented to being contacted for future research were identified and participants were then recruited randomly. The presented results are based on data collected at the first neuroimaging assessment. Ethical approval was obtained from the CHU Sainte-Justine Research Ethics Committee in Montreal. All participants actively assented or consented to the study procedures, and parent consent was obtained for minors.

### Exclusion criteria

Exclusion criteria for the Pro-Venture study included DSM-5 psychiatric disorders, family history of schizophrenia, taking antipsychotic medication, neurological disorders or IQ < 70 and a contraindication to undergo MRI examination. Prior to neuroimaging acquisition, participants were administered a urinary test for the recent consumption of substances and participants who had used alcohol, cannabis, MDMA, cocaine, or opioids did not undergo neuroimaging. Since cannabis metabolites can be detected up to 7 days following its sporadic use, individuals who tested positive but reported not having consumed cannabis within 24 h (and did not display signs of intoxication) prior to neuroimaging were invited.

### Clinical assessments

PLEs (hallucinations, delusional beliefs, suspiciousness, strange experiences, and feelings of grandiosity) in the last 12 months were assessed with the *Adolescent Psychosis Screening Scale* (APSS) using nine items that have previously been described^9^. Three questions from the questionnaire were shown to have significant positive predictive power for interview-verified PLEs that ranged between 80 and 100%^43^. Psychotic symptoms were measured using the CAPE^44^. Substance use during the past 12 months was measured using the DEP-ADO questionnaire^45^. Participants rated their consumption of alcohol, tobacco, cannabis, and other substances on a scale of 6 levels, ranging from “never” to “everyday”^46^. More detailed accounts of quantity and frequency of substance use were also collected using the timeline follow-back method^47^ for the 4 weeks prior to assessment. The Development and Well-Being Assessment (DAWBA) package was used to generate ICD-10 and DSM-IV psychiatric diagnoses^48^. Externalizing and internalizing behaviors were evaluated with the Strength and Difficulties Questionnaire (SDQ)^49^. Finally, the Penn Emotion Recognition task ER-40 (ref. ^50^) was used to assess facial emotion recognition ability.

### Task design

We employed a task-based fMRI face recognition paradigm derived from Grosbras and Paus^51^ which was used by the IMAGEN consortium in youths with PLEs^18^. The task consisted of short sequences (2–5 s) of black and white videoclips of dynamic facial expressions. Compared to static emotional faces, dynamic facial expressions are more representative of social interactions and have been shown to elicit larger activations in brain areas involved in social and emotional processing^51–53^. Three categories of videoclips were presented: (1) actors portraying neutral facial expressions turning into angry expressions, (2) actors portraying neutral facial expressions turning into different neutral expressions, and (3) a control condition consisting of a dynamic video of expanding/contracting circles. The control condition was designed to roughly match the physical properties of the dynamic facial expressions in terms of color, light, contrast, and motion characteristics.

### Neuroimaging acquisition parameters

Blood oxygenated level dependent (BOLD) signal was acquired at the Unité de Neuroimagerie Fonctionnelle de l’Institut de Gériatrie de l’Université de Montréal on a Siemens Prisma Fit 3T scanner. Functional imaging data were acquired with a T2-weighed multiband echo planar imaging sequence (TR = 785 ms; TE = 30 ms; FA = 54°; matrix size 64 × 64, voxel size 3 mm^3^; 42 slices). Functional slices were oriented in transverse plane and were angled to be parallel to the AC-PC line. During EPI image acquisition, an inline retrospective motion correction algorithm was employed. During the same session, high-resolution T1-weighted anatomical images were acquired (TR = 2300 ms; TE = 2.98 ms; FA = 9°; matrix size=256 × 256; voxel size = 1 mm^3^; 176 slices).

### fMRI preprocessing

Preprocessing was conducted using the CONN-fMRI functional connectivity toolbox (www.nitrc.org/projects/conn)^54^. Functional images were realigned, corrected for motion artifacts with the *Artifact Detection Tools* (ART), high pass filtered (>0.008 Hz) and co-registered to the corresponding anatomical image. Outliers were detected by scanning the global BOLD signal and removing volumes associated to signal changes above 4 standard deviations, or framewise displacements above 0.5 mm. The anatomical images were segmented into gray matter, white matter, and cerebrospinal fluid. Functional images were then normalized to Montreal Neurological Institute stereotaxic space using the deformation field from the corresponding anatomical images and were smoothed with a 6-mm 3D isotropic Gaussian kernel. The anatomical component-based noise correction method (CompCor)^55^ was used as it removes confounding effects from the BOLD timeseries, such as the physiological noise originating from the white matter and cerebrospinal fluid^56^.

### Brain activation analyses

A general linear model (GLM) was mapped for each participant using SPM12 (https://www.fil.ion.ucl.ac.uk/spm/software/spm12/)^57^ in Matlab version 2020a. The experimental conditions of Angry (angry facial expressions), Neutral (neutral facial expressions), and Control (expanding circles) were convolved to a canonical hemodynamic response function. The “FAST” model was implemented to account for temporal correlations^58^. Our primary contrast of interest was defined as [(Angry+Neutral) − Control] and isolates facial cue processing. We also implemented two other contrasts: the (Angry − Neutral), which isolates negative emotion processing, and the (Neutral − Control) contrast, which isolates neutral facial processing. Between-group differences were evaluated using a full analysis of variance (ANOVA) *F*-test comparing the three groups for each of the three contrasts of interest, using subject as a random effect. Estimated parameters of the regressors in the GLM model (beta weights) were extracted using the Marsbar toolbox^59^. The statistical threshold for significance was determined using a Monte Carlo simulation^60^. Assuming a per probability of *p* < 0.001, after 10,000 simulations, a cluster size of 21 resampled voxels was indicated to correct for multiple comparisons at *p* < 0.05. Considering the amygdala’s key role in emotion processing, we further used a small volume correction approach to perform a region of interest analysis^61^.

### Functional connectivity analyses

Voxel-wise seed-based functional connectivity analyses were performed in CONN. To examine differences in task-related connectivity between groups, generalized psycho-physiological interaction (gPPI) analyses were performed. The gPPI model generated a regressor of each condition (Angry, Neutral, and Control), representing the level of task-modulated functional connectivity between the seed and every voxel in the brain. This approach has been shown to generate a more accurate model of the interaction between the conditions and the neural activity^62^. The model includes a psychological regressor for each condition (the task effects convolved with a canonical hemodynamic response function), a physiological regressor (the seed’s BOLD timeseries), and an interaction term consisting of the product of the psychological and physiological terms. The psychophysiological interaction term was then modeled against the time course for all voxels in the brain to test for significant connectivity effects. Seed regions were defined based on the regions that significantly differed between groups during the activation analyses. Given its role in threat-perception and negative emotion processing, the bilateral amygdalae, as defined in the Harvard-Oxford atlas, were also used as seeds of interest. Between-group differences were evaluated using a full ANOVA for each of the selected contrasts. The peak threshold was set at *p* < 0.001 with a cluster threshold of *p* < 0.05 corrected for the false discovery rate. Beta weights were extracted for all significant findings. Post hoc analyses using beta weights were performed with and without Bonferroni correction for the number of seeds being tested.

### Statistical analyses

Between-group differences in continuous data were examined with ANOVAs. Post hoc analyses were performed using Tukey’s Honestly significant difference test. For dichotomous data, *χ*^2^ tests and pairwise comparisons were performed. Significance was set at *p* < 0.05. Analysis of covariance assessed effects of clinical variables on the between-group differences in brain activity and connectivity. Bivariate Pearson correlation analyses were performed to determine the relationship between clinical variables (e.g., CAPE-42 subscales) and activation and connectivity strength estimates across groups (*p* ≤ 0.05, Bonferroni corrected).

## Supplementary information

Supplemental material

## Data Availability

The data are not publicly available as they contain information that could compromise research participant privacy/consent. The data that support the findings of this study are available upon reasonable request from the corresponding author, S.P., but are only redistributable to researchers engaged in IRB approved research collaborations.
