# Supplementary material for: Neural alterations of emotion processing in atypical trajectories of psychotic-like experiences
Source: Schizophrenia (Heidelb). 2022 Apr 21;8(1):40. doi: 10.1038/s41537-022-00250-y (PMC9261083; doi:10.1038/s41537-022-00250-y)
Supplement: Supplementary file 1 — Supplemental material [file 41537_2022_250_MOESM1_ESM.docx]

**Supplement 1:**

**Determination of PLE trajectories**

Previously, a latent-class growth model was used to estimate the PLE trajectories in the Co-Venture sample of over 2500 youths using four-year annual PLE data between ages 13 to 16 ^1^. In short, group-based trajectories were estimated using growth mixture models which were then fitted with different models ranging from one to four trajectories. The best-fitting model was determined to be the three-trajectory model using the Bayesian Information Criterion (BIC), the Akaike Information Criterion (AIC), the Lo-Mendell-Rubin Likelihood Ratio Test (LMR-LRT), and entropy. Missing data on the dependent variable (PLE score) were handled through Full Information Maximum Likelihood.

**Supplement 2:**

**Connectivity maps**

**
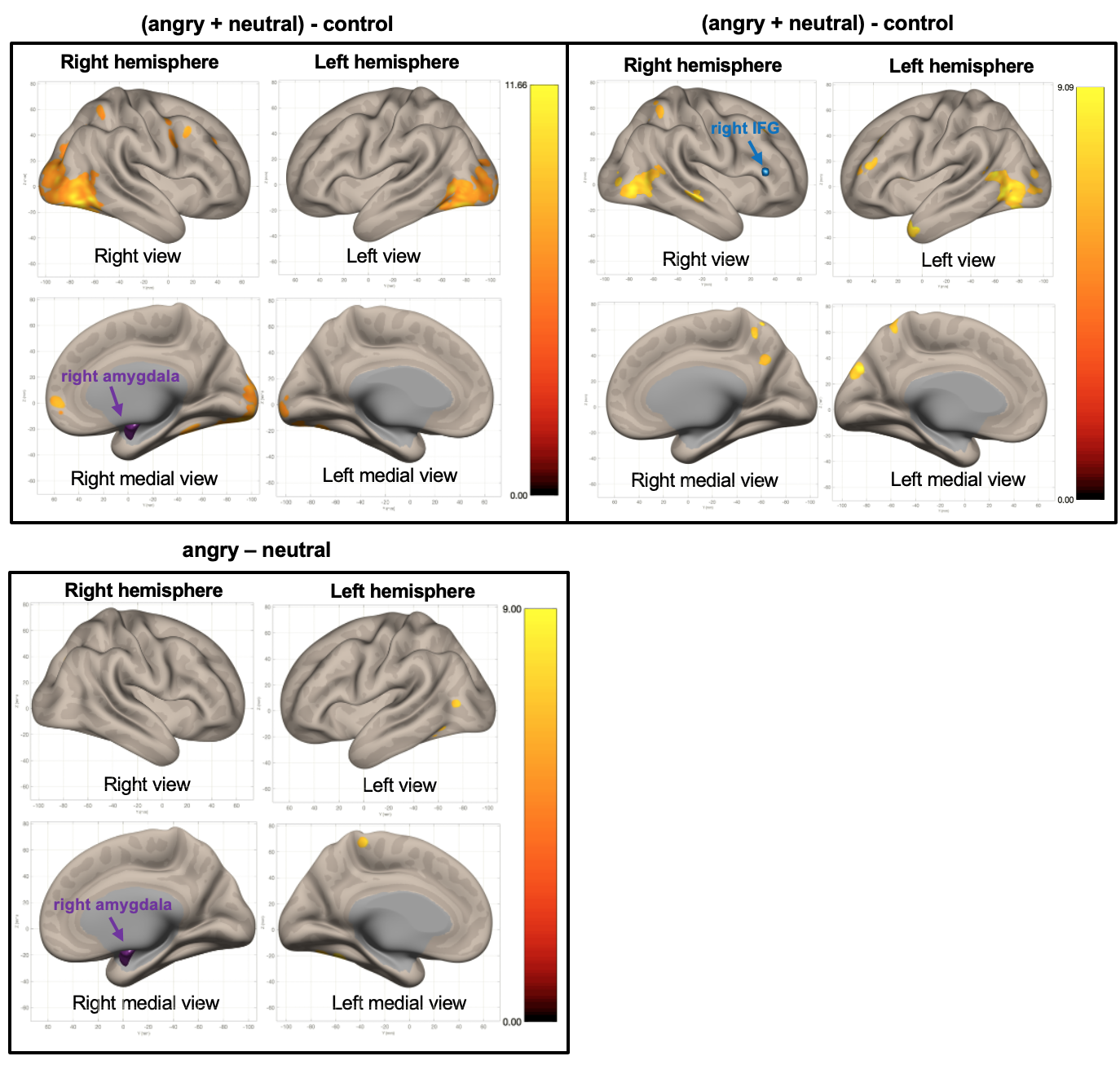
**

Between-group connectivity maps of the significant connectivity results reported in Figure 2, showing the relevant seeds (the right amygdala and the right inferior frontal gyrus) and the corresponding voxels during the various contrasts. In the [(Angry + Neutral) – Control] facial cue processing contrast, the right amygdala exhibits significantly altered connectivity with the right ventromedial prefrontal cortex, and the right inferior frontal gyrus displays significantly altered connectivity with the right middle temporal cortex, the right precuneus, and the left temporopolar cortex. In the (Angry – Neutral) negative emotion processing contrast, the right amygdala exhibits significantly altered connectivity with the right parietal angular cortex, the left visual association cortex, and the left cerebellum (not shown here).

**Supplemental references**

1. Bourque, J., Afzali, M. H., O’Leary‐Barrett, M. & Conrod, P. Cannabis use and psychotic-like experiences trajectories during early adolescence: the coevolution and potential mediators. *Journal of Child Psychology and Psychiatry* **58**, 1360–1369 (2017).
